# Idiopathic Bell’s Palsy in a Five-Week-Old Infant: A Case Report

**DOI:** 10.7759/cureus.77622

**Published:** 2025-01-18

**Authors:** Ethan Dimock, Usha Kellampalli, Wendy Wismer, Alise Haddad, Bassel Salman

**Affiliations:** 1 Medicine, Oakland University William Beaumont School of Medicine, Rochester, USA; 2 Internal Medicine and Pediatrics, Corewell Health William Beaumont University Hospital, Royal Oak, USA; 3 Pediatrics, Corewell Health William Beaumont University Hospital, Royal Oak, USA

**Keywords:** bell`s palsy, cranial nerve vii palsy, infant case report, pediatric neurology, pediatric otolaryngology

## Abstract

Bell’s palsy is a paralysis on one side of the face due to inflammation of the seventh cranial nerve and most often has an idiopathic etiology. This case presents a five-week-old male who was noted to have a left-sided facial asymmetry. Pathological etiologies were ruled out, but the findings were inconclusive, and the patient was ultimately discharged on a course of oral prednisolone. This report highlights the youngest documented case of idiopathic Bell’s palsy, detailing the diagnostic challenges and successful treatment approach that led to complete recovery.

## Introduction

Bell’s palsy is most often of idiopathic origin and is a diagnosis of exclusion, hence why it is also given the moniker "acute facial palsy of unknown cause" [1]. The paralysis is rapid in onset and involves partial or complete weakness of one half of the face [2]. Other potential associated symptoms include alterations in taste, lacrimation, salivation, and sensitivity to sound [2]. Although no age is completely immune to this condition, the median age of onset is 40 years [1,2]. The annual incidence of facial nerve palsy is approximately 2.7 per 100,000 in children under 10 years old and increases to an estimated 10.1 per 100,000 in those aged 10 to 20 years [3].

## Case presentation

We are presenting a five-week-old Caucasian male patient with left-sided facial asymmetry. This patient was born full-term at 39 weeks via an elective C-section and had no perinatal complications. The only remarkable finding during prenatal care was a stage II hydronephrosis observed on ultrasound.

He was in his normal state of health until his mother noticed at five weeks of life that “his left eye would not close when he was crying.” She also noticed that his left nasolabial fold was flattened and that the facial palsy persisted throughout the day. Before the presentation, the parents reported no feeding, stooling, or voiding issues. He also did not have any fevers or fussiness before arrival. There was also no facial injury at birth or in the four following weeks.

The patient was exposed to COVID-19 via his grandparents, who tested positive about two weeks prior. However, no upper respiratory symptoms were observed, and he was feeding well.

The workup included a hepatic function panel, complete blood count, prothrombin time and partial thromboplastin time, and basic metabolic panels, which were all grossly normal. Additionally, the screening tests for COVID-19, influenza A and B, respiratory syncytial virus, meningitis, and the encephalitis panel were all unremarkable. A lumbar puncture was also performed, and the results were negative.

**Figure 1:**
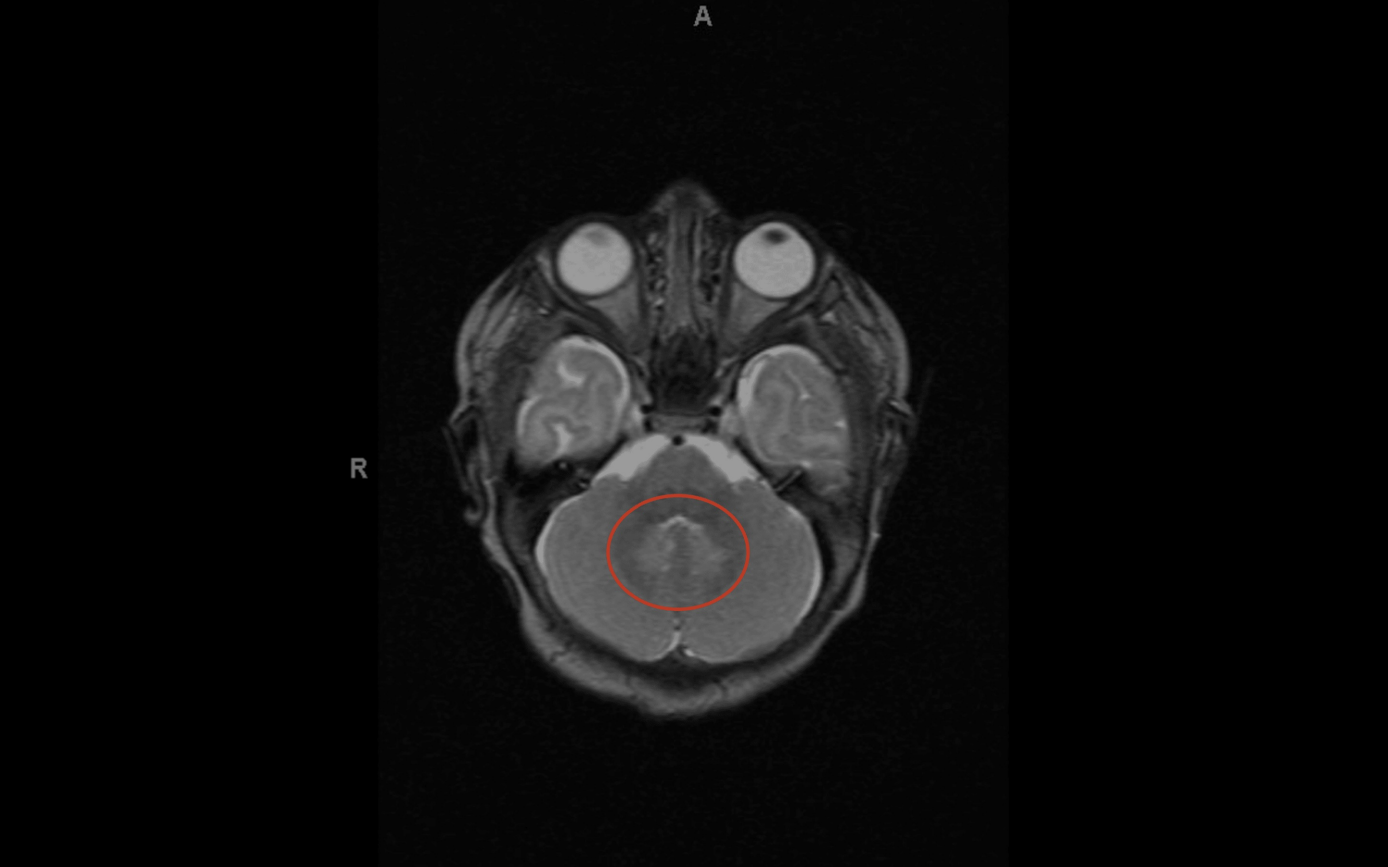
Symmetric patchy T2 hyperintense signal changes in bilateral dentate nuclei (red circle)

Ancillary imaging was performed, and the brain MRI demonstrated a symmetric T2 hyperintensity in bilateral dentate nuclei without diffusion restriction or pathologic enhancement (Figure 1). The findings were nonspecific and can be seen in multiple conditions that involve inflammation, infection, metabolic dysfunction, toxin exposure, and the adverse effects of drugs. No other significant abnormalities were seen in the brain. After discussing the findings with the radiologist, it was deemed that these findings were unrelated to the facial asymmetry.

The patient was started on daily oral prednisolone at a dose of 1 mg/kg/dose, twice-daily erythromycin ophthalmic ointment, and intravenous acyclovir at a dose of 20 mg/kg/dose for possible herpes simplex virus-induced Bell’s palsy. Acyclovir was discontinued once the herpes simplex virus polymerase chain reaction was shown to be negative.

The patient was discharged with a total of seven days of oral prednisolone (two of which he had already completed in the hospital). With the completion of the treatment regimen, he initially displayed a recovery of function in the lower face, followed by a subsequent recovery of function in the upper face a few days later, resulting in a complete recovery.

## Discussion

Since the median age of presentation for Bell’s palsy is 40 years [2], it is rare to see this presentation in infants. Risk factors include diabetes, hypertension, pregnancy, obesity, and upper respiratory tract infections [1]. There is no reported case of Bell’s palsy in a five-week-old infant.

The most common cause of seventh cranial nerve palsy in this age group is related to birth trauma [4]. Our patient had a normal newborn exam at birth and displayed no abnormalities at his two- and four-week follow-ups.

There have been a few cases of children who experienced an acute onset of Bell's palsy. The youngest patient described was a previously healthy three-year-old who was diagnosed with acute-onset idiopathic Bell’s palsy [5]. Another case described a five-month-old infant who was also diagnosed with idiopathic facial nerve palsy, although this case was associated with upper respiratory symptoms [6]. These symptoms included rhinorrhea, cough, and nasal congestion. The patient that we presented did not have any associated symptoms. The case most similar to ours involved a 15-day-old infant with no birth complications and who was previously healthy, who was eventually diagnosed with Bell’s palsy after all other potential etiologies were ruled out [7].

Since the cause of facial paralysis is usually unclear on initial presentation, especially in this age group, the condition is treated empirically with a combination of antiviral drugs and corticosteroids [8]. This combination has been shown to have better long-term outcomes than when the patient is treated with prednisolone alone [8]. The rationale behind the antiviral drug therapy is that until one of the most devastating potential causes of Bell’s Palsy, human herpesvirus, is ruled out through imaging and lab testing, the patient must be treated as though they are human herpesvirus-positive. Despite the existing body of evidence, the patient, in this case, was in uncharted territory regarding this empiric treatment because the evidence is based on research involving three- and six-month-old children, whereas this child was only five weeks of age. Nonetheless, the patient was treated according to these guidelines, as they represent the best existing evidence for managing a case like this.

Additionally, Bell’s palsy in infants can pose diagnostic challenges due to the overlap in presentation with other neurological conditions. Although rare, Lyme disease has been reported as a cause of facial nerve palsy in children, particularly in endemic regions, necessitating careful consideration of travel and exposure history during the evaluation process [9]. Additionally, congenital conditions such as Moebius syndrome can present with bilateral facial paralysis, further complicating the diagnosis [10]. In cases where the diagnosis is uncertain, imaging studies like MRI can help identify structural or inflammatory causes, distinguishing Bell’s palsy from other conditions.

## Conclusions

This is the only documented case of idiopathic Bell’s palsy for a patient in this age group. Although several diagnostic imaging and laboratory tests were performed to ascertain a diagnosis, none of these modalities revealed any remarkable findings; thus, the patient was treated empirically. Ultimately, he made a full recovery and returned to his baseline.
